# The effect of obesity and subsequent weight reduction on cardiac structure and function in dogs

**DOI:** 10.1186/s12917-022-03449-4

**Published:** 2022-09-20

**Authors:** C. Partington, H. Hodgkiss-Geere, G. R. T. Woods, J. Dukes-McEwan, J. Flanagan, V. Biourge, A. J. German

**Affiliations:** 1 Institute of Infection, Veterinary, Ecological and Sciences, Department of Small Animal Clinical Sciences, Teaching Hospital, University of Liverpool, Chester High Road, Neston, CH64 7TE Wirral UK; 2 Present address: Department of Veterinary Medicine, University of Cambridge, Madingley Road, Cambridge, CB3 0ES UK; 3 Institute of Life Course and Medical Sciences, Department of Small Animal Clinical Sciences, Teaching Hospital, University of Liverpool, Chester High Road, Neston, CH64 7TE Wirral UK; 4 Royal Canin Research Center, 650 Avenue de la petite Camargue – CS10309, 30470 Aimargues, France

**Keywords:** Heart rate variability, Cardiac biomarkers, Canine, Echocardiography

## Abstract

**Background:**

In people, the cardiovascular effects of obesity include systemic hypertension, cardiac remodelling and both systolic and diastolic dysfunction, whilst weight reduction can reverse myocardial remodelling and reduce risk of subsequent cardiovascular disease. To date, variable results are reported in studies of the effect of obesity and controlled weight reduction on cardiovascular morphology and function in dogs. This prospective study aimed to assess cardiac function, heart rate variability, cardiac biomarkers and body composition before and after weight reduction in pet dogs with obesity. Twenty-four client-owned dogs referred for weight management due to obesity were recruited. To assess the cardiac effects of obesity, body composition analysis (by dual energy X-ray absorptiometry, DEXA) and cardiovascular assessment (echocardiography, Doppler blood pressure, electrocardiography, cardiac biomarkers) were performed prior to weight management. Twelve dogs completed the study and reached target weight, receiving a further cardiovascular assessment and DEXA. A Wilcoxon-signed rank test was used to compare each variable pre- and post- weight reduction.

**Results:**

Median (interquartile range) duration of weight loss was 224 days (124–245 days), percentage weight loss was 23% (18–31%) of starting weight. Median change in body fat mass was -50% (-44% to -55%; *P* = 0.004), whilst median change in lean mass was -7% (+ 1% to -18%, *P* = 0.083).

Before weight reduction, diastolic dysfunction (evidence of impaired relaxation in all dogs), increased left ventricular wall thickness and mildly elevated systolic blood pressure (14/24 ≥ 160 mmHg, median 165 mmHg (140–183)) were common features in dogs with obesity. However, systolic left ventricular wall dimensions were the only variables that changed after weight reduction, with a decrease in both the systolic interventricular septum (*P* = 0.029) and systolic left ventricular free wall (*P* = 0.017). There was no evidence of decreased heart rate variability in dogs with obesity (*P* = 0.367), and no change in cardiac biomarker concentrations with weight reduction (N-terminal proBNP, *P* = 0.262; cardiac troponin I *P* = 0.657).

**Conclusions:**

Canine obesity results in diastolic dysfunction and left ventricular hypertrophy, the latter of which improves with significant weight and fat mass reduction. Further studies are required to clarify the clinical consequences of these findings.

**Supplementary Information:**

The online version contains supplementary material available at 10.1186/s12917-022-03449-4.

## Introduction

Obesity in pet dogs is a common, major health concern that has been associated with an increase in morbidity and shorter median lifespan [1, 2]. In humans, it is an independent cardiovascular risk factor, mainly due to its association with atherosclerosis and ischaemic myocardial disease [3, 4]. Obesity is also associated with chronic volume overload, increased cardiac output, and activation of both the renin–angiotensin–aldosterone system (RAAS) and sympathetic nervous system. However, variable findings are evident in reports about the resultant myocardial morphological and functional effects of obesity in people. Left ventricular (LV) hypertrophy is commonly reported, with or without concurrent chamber dilation. Impairment in both systolic and diastolic function is also reported, the latter being more common [4].

Similar effects are also reported in dogs with obesity, although with some variable findings between reports. Mehlman et al. [5] reported an increase in systolic blood pressure (SBP), hypertrophy of the left ventricular free wall (LVFW) in both diastole and systole and reduced diastolic function in dogs with obesity. In contrast, Adolphe et al. [6] reported only the systolic, not diastolic, thickness of the LVFW to be increased in canine obesity, along with a clinically irrelevant increase in SBP (diastolic function was not assessed). Conversely, Tropf et al. [7] reported hypertrophy of the interventricular septum (IVS) in diastole, with no change in the LVFW, in dogs with obesity; reduced diastolic function but no significant difference in SBP was also reported.

Reversal of structural and haemodynamic abnormalities associated with human obesity has been demonstrated with weight reduction, with reduced LV mass and improved diastolic function [8, 9]. A decrease in LV mass has been reported following weight reduction in some canine studies [6, 10, 11]; however, changes in diastolic function have only been assessed in one of these studies [11].

Despite the gross cardiac changes associated with obesity in various species, it remains unclear whether these structural changes are merely compensatory or reflect deteriorating myocardial function which may impact exercise capacity and quality of life. Furthermore, it remains uncertain whether such changes in myocardial function improve with weight reduction in dogs.

Echocardiographic Tissue Doppler imaging (TDI) is used to evaluate myocardial motion and is sensitive at identifying subtle changes in systolic and diastolic function. TDI has been used to identify both systolic and diastolic dysfunction in humans with obesity, prior to detectable changes in more conventional echocardiographic parameters [12, 13]. Identifying such changes in advance of the development of overt abnormalities, in otherwise healthy patients with obesity, might be important in explaining myocardial dysfunction which may later lead to increased morbidity and mortality [14]. TDI has been used in assessment of diastolic dysfunction in dogs with obesity [7]; however, as far as the authors are aware, pulsed wave TDI (PW-TDI) has not been used to investigate the effect of subsequent weight reduction on diastolic function in dogs.

Obesity is associated with an increase in heart rate in people, in part due to altered sympathovagal balance [4]. Heart rate variability (HRV) is an indicator of this autonomic tone and has been used to predict risk of cardiovascular disease in people with obesity [15]. There are few studies on HRV in dogs with obesity. Pongkan et al. [16] reported reduced HRV in a small cohort of male dogs with obesity, similar to the findings of Vieira et al. [17], who also reported reduced HRV in a small cohort of mild to moderately overweight dogs. However, to the authors’ knowledge, changes in HRV in dogs following weight reduction have not previously been assessed.

We hypothesised that dogs with obesity would show signs of systolic and diastolic dysfunction, increased LV wall thickness and reduced heart rate variability, all of which may improve with weight reduction. Therefore, the first aim of the current study was to assess a cohort of dogs with obesity for the presence of systolic dysfunction, diastolic dysfunction and altered LV wall thickness. The second aim was to monitor for changes in echocardiographic variables in response to a controlled weight reduction programme, utilising dual energy x-ray absorptiometry (DEXA), to quantify changes in body composition. A final aim was to monitor for changes in autonomic balance by examining changes in HRV during this controlled weight reduction programme.

## Results

### Study animals

Twenty-four dogs met the initial inclusion criteria and were enrolled in the study; of these only 12 achieved target weight reduction and were included in final analysis. A variety of breeds were represented (Supplement Table 1), with a median age of 67 months (interquartile range [IQR] 41mo—101mo) at time of enrolment. Median weight for all dogs at enrolment was 14.6 kg (IQR 11.03–41.2 kg) with a median body condition score (BCS) of 8/9 (IQR 7/9 – 9/ 9). There were 14 females (three sexually-intact, 11 neutered) and 10 males (two sexually-intact, eight neutered). One dog did not undergo DEXA pre- or post-weight-reduction due to lack of consent for sedation. There was no difference in the age (*P* = 0.214, r = 0.333), bodyweight (*P* = 0.665, *r* = 0.133) or BCS (*P* = 0.330, *r* = 0.290) at time of inclusion between those dogs that did and did not achieve the target bodyweight.

**Table 1:** Weight reduction and dual energy x-ray absorptiometry (DEXA) data for the dogs that achieved target weight reduction

Variable	Before weight reduction		After weight reduction		*P* value	*r value*
	**Median**	**(IQR)**	**Median**	**(IQR)**		
**Weight (kg)**	19.7	(11.55–36.07)	15.40	(8.22–25.85)	**0.003**	0.884
**BCS (/9)**	8	(7–8)	5	(4–5)	**0.004**	0.895
**Body fat (%)**	40.36	(37.52–48.27)	25.63	(21.9–33.11)	**0.004**	0.847
**Body fat (g)**	8224	(4657–14,514)	4518	(2106–8164)	**0.004**	0.885
**Lean mass (g)**	12,849	(6084–18,505)	11,961	(5109–16,991)	0.083	0.402
**BMC (g)**	637	(253–1182)	594	(209–893)	**0.004**	0.885

*BCS* Body condition score, *BMC* Bone mineral content, *IQR* Interquartile range

### Baseline cardiovascular variables (all dogs)

The baseline data for cardiovascular variables for all dogs is shown in Supplement Tables 1 and 2. N-terminal Pro B-type Natriuretic Peptide (NT-proBNP) was below the laboratory reference range of 900 pmol/L in all dogs at baseline (median 285 pmol/L, IQR 250–377) excluding significant increased myocardial wall stress. Baseline high-sensitivity cardiac troponin I (hs-cTnI) was missing for one dog; one dog had a moderately increased hs-cTnI at baseline (1.5 ng/mL, reference interval [RI]: < 0.07), echocardiography of this dog showed stage B1 myxomatous mitral valve disease (MMVD), mild hypertrophy of the IVS and LVFW and subjective volume depletion; Doppler blood pressure was 180 mmHg (assumed to be stress induced due to patient anxiety). Cardiac troponin I for all other dogs at baseline was normal (median 0.009 ng/mL, IQR 0.005- 0.015). Median vasovagal tonus index (VVTI) was 8.34 (IQR 6.70–10.31). Fourteen of the twenty-four dogs (58%) had elevated SBP (BP ≥ 160 mmHg) on enrolment (median 165 mmHg, IQR 140–183).

**Table 2:** Cardiovascular and echocardiographic variables pre- and post- weight reduction for the dogs that achieved target weight reduction

Variable		Before weight reduction		After weight reduction		*P* value	**r value**
		**Median**	(IQR)	**Median**	(IQR)		
**ECG heart rate**		115	(95- 140)	110	(88.75–137.5)	0.722	0.117
**Heart rate variability (VVTI)**		8.337	(6.7- 10.1)	8.856	(7.69–10.13)	0.367	0.272
**SBP (mmHg)**		178	(140–184)	148	(136.3–188.8)	0.554	0.164
**Hs-cTnI (ng/mL)**		0.013	(0.007–0.52)	0.012	(0.009–0.018)	0.657	0.147
**NT-proBNP (pmol/L)**		371.5	(257.8–530.0)	482.0	(345.0–558.0)	0.262	0.353
**LA/Ao**		1.305	(1.14–1.44)	1.295	(1.13–1.37)	0.894	0.013
**LA major (cm)**		3.4	(2.78–3.97)	3.335	(2.87–3.94)	0.722	0.125
**LAmax/Ao**		2.3	(2.06–2.42)	2.1	(1.75–2.29)	0.176	0.436
**IVSd (mm)**		10.25	(8.93–12.25)	9.65	(7.88–10.7)	0.158	0.419
**LVIDd (mm)**		35.2	(27.43–42.25)	34.45	(27.27–42.18)	0.556	0.363
**LVFWd (mm)**		9.3	(8.28- 10.88)	8.8	(7.9–9.48)	0.099	0.340
**IVSs (mm)**		13.4	(11.85–15.9)	11.75	(9.65–14.1)	**0.029**	0.643
**LVIDs (mm)**		23.75	(17.23–31.27)	25.45	(18.97–27.93)	0.272	0.323
**LVFWs (mm)**		13.3	(12.1–15.5)	10.55	(10.15–14.3)	**0.017**	0.702
**IVSdN**	**Actual weight**	0.41	(0.38–0.47)	0.41	(0.38–0.48)	0.610	0.158
	**Target weight**	0.46	(0.41–0.55)	0.41	(0.38–0.49)	0.126	0.453
**LVIDdN**	**Actual weight**	1.31	(1.18–1.41)	1.41	(1.23–1.53)	0.092	0.498
	**Target weight**	1.44	(1.32–1.60)	1.42	(1.26–1.54)	0.290	0.317
**LVFWdN**	**Actual weight**	0.44	(0.39–0.51)	0.42	(0.37–0.50)	0.610	0.158
	**Target weight**	0.49	(0.43–0.55)	0.42	(0.37–0.51)	0.196	0.385
**IVSsN**	**Actual weight**	0.58	(0.49–0.68)	0.55	(0.48–0.59)	0.224	0.362
	**Target weight**	0.63	(0.55–0.74)	0.56	(0.50–0.60)	**0.011**	0.744
**LVIDsN**	**Actual weight**	0.82	(0.72–0.93)	0.94	(0.81–1.04)	**0.011**	0.747
	**Target weight**	0.91	(0.81–1.04)	0.95	(0.83–1.05)	0.196	0.385
**LVFWsN**	**Actual weight**	0.66	(0.54–0.71)	0.59	(0.54–0.64)	0.196	0.385
	**Target weight**	0.72	(0.58–0.79)	0.60	(0.54–0.65)	**0.009**	0.770
**EDV (mL)**		32	(18–65)	33	(18–64)	0.507	0.200
**EDVI (mL/kg)**	**Actual weight**	1.67	(1.21–2.09)	2.02	(1.88–2.72)	**0.008**	0.770
	**Target weight**	2.31	(1.80–2.59)	2.01	(1.88–2.72)	0.657	0.128
**ESV (mL)**		13	(6–38)	13	(7–35)	0.180	0.404
**ESVI (mL/kg)**	**Actual weight**	0.64	(0.42–1.13)	0.92	(0.67–1.41)	**0.015**	0.702
	**Target weight**	0.90	(0.65–1.43)	0.92	(0.67–1.41)	0.158	0.407
**E velocity (m/s)**		0.67	(0.56–0.76)	0.71	(0.53–0.79)	0.965	0.026
**MV E/A**		1.175	(0.9–1.40)	1.03	(0.88–1.24)	0.824	0.119
**E deceleration (ms)**		90	(77.75–108)	86.5	(81.75–106.5)	0.76	0.103
**IVRT (ms)**		73.5	(62.5–87.75)	75	(69–94.25)	0.724	0.113
**TAPSE (cm)**		1.14	(0.9–1.5)	1.11	(0.8–1.36)	0.789	0.309
**Septal E’/A’**		0.695	(0.57–0.83)	0.7	(0.59–0.84)	0.45	0.242
**Lateral E’/A’**		0.715	(0.59–0.80)	0.7	(0.6–0.8)	0.824	0.081
**Right E’/A’**		0.65	(0.6–0.86)	0.67	(0.56–0.72)	0.262	0.371
**Fractional shortening (%)**		32	(27–39)	30	(26–34)	0.168	0.411
**Ejection Fraction (%)**		61	(53–65)	56	(41–69)	0.266	0.349

*EDV* End diastolic volume, *EDVI* End diastolic volume index, *ESV* End systolic volume, *ESVI* End systolic volume index, *hs-cTnI* Cardiac troponin I, suffix: -N: indexed to bodyweight, *IQR* Interquartile range, *IVRT* Isovolumetric relaxation time, *IVSd* Interventricular septum in diastole, *IVSs* Interventricular septum in systole, *LA/Ao* Left atrium: aorta ratio, *LAmax/Ao* Left atrium major to aorta ratio, *LVFWd* Left ventricular free wall in diastole, *LVFWs* Left ventricular free wall in systole, *LVIDd* Left ventricular internal diameter in diastole, *LVIDs* Left ventricular internal diameter in systole, *MV* Mitral valve, *NT-proBNP* N-type N-terminal pro-brain natriuretic peptide, *SBP* Blood pressure, *TAPSE* Tricuspid annular plane systolic excursion, *VVTI* Vasovagal tonal index

Eight dogs were diagnosed with stage B1 MMVD, one dog was diagnosed with mild/equivocal aortic stenosis (aortic velocity 2.56 m/s, RI: < 2.25; pressure gradient 26 mmHg). First-degree atrio-ventricular (AV) block was suspected during echocardiography for one dog; its six-lead electrocardiography (ECG) confirmed both first degree, and intermittent Mobitz type I second degree AV block and an intraventricular conduction disturbance (left anterior fascicular block). One dog was in sinus tachycardia throughout echocardiography with a heart rate of 160 bpm on six-lead ECG (Supplement Table 1). Two dogs had mild left atrial (LA) enlargement based on two-dimensional (2D) diastolic left atrium/aorta ratio (LA/Ao), all remaining dogs had normal left atrial size (median LA/Ao 1.28, IQR 1.13–1.38, RI: < 1.5). When normalised to target bodyweight [18], six/twenty-four dogs (25%) had an IVS-diastole above reference range (median 0.45, IQR 0.40–0.51, RI: 0.27–0.49), four/twenty-four dogs (17%) had a LVFW-diastole above reference range (median 0.47, IQR 0.41–0.51, RI: 0.3–0.53); four/twenty-four (17%) and three/twenty-four (13%) had an IVS-systole (median 0.56, IQR 0.50–0.60, RI: 0.38–0.68) and a LVFW-systole (median 0.60, IQR 0.54–0.65, RI: 0.46–0.78) above reference range respectively.

There was evidence of diastolic dysfunction with Tissue Doppler imaging of both the lateral and septal LV walls consistent with impaired relaxation in all dogs (E’/A’ < 1); transmitral flow showed an impaired relaxation pattern in 7/24 (29.2%; mitral E/A < 1), increased isovolumetric relaxation time in 18/24 (75%; median 76.0 ms, IQR 67.0–84.0, RI 37–69) and increased E deceleration time in 5/24 (20.8%; median 86.0 ms, IQR 75.0–102.0, RI 52–108). There was also evidence of systolic dysfunction based on reduced fractional shortening (< 25%), Simpson’s derived ejection fraction (< 50%) and end-systolic volume index (ESVI; > 1.54 mL/kg) in 3/24 (12%), 4/24 (17%) and 1/24 (4%) respectively. Of these, five dogs had a reduction in only one variable of systolic function, with normal left ventricular dimensions on allometric scaling; one had reduced systolic function based on all three variables but normal left ventricular dimensions. Subjectively none of the dogs with reduced systolic function were suspected to have dilated cardiomyopathy (Supplement Table 1) and no dog had an increased diameter to wall ratio (left ventricular internal diameter in diastole / LVFW in diastole [LVIDd/LVFWd], RI: 2.9–6.7 [19]).

### Weight reduction

Weight reduction data for the 12 dogs that completed the study is shown in Table 1. Weight reduction was achieved over a median time of 224 days (IQR 124–245 days). Median body weight reduction was 4.55 kg (IQR 3.2–6.57), equating to a decrease in body weight of 23% (IQR 18–31%). Body condition score decreased by a median of 3 units (IQR 2–4, *P* = 0.003, r = 0.895), lean mass (g) changed by -7% (IQR + 1 to -18, P = 0.083, r = 0.402) and body fat percentage changed by -50% (IQR -44 to -55, *P* = 0.004, r = 0.847).

### Changes in cardiovascular parameters with weight reduction

**Fig. 1:**
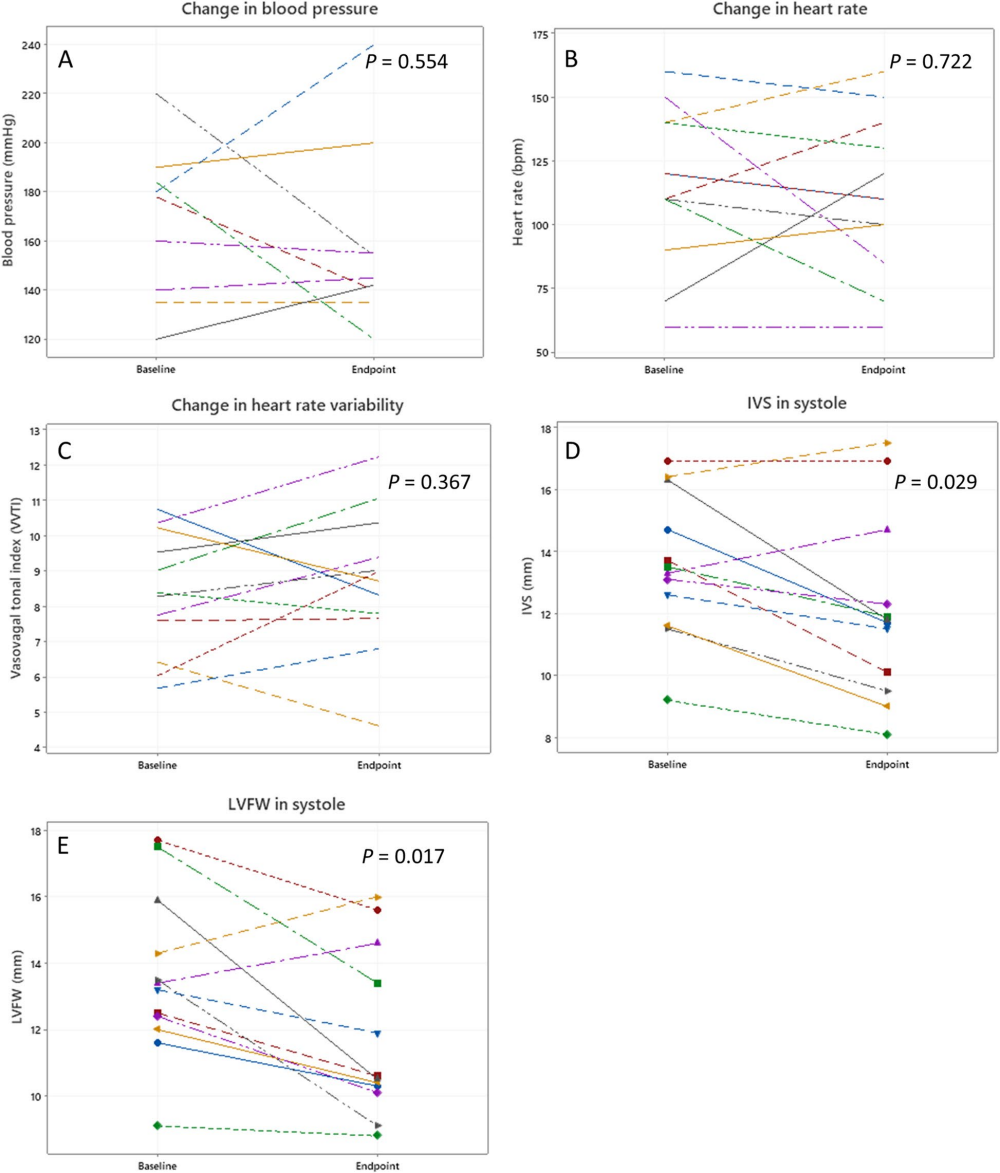
Changes in cardiovascular variables with weight reduction. Line plots showing the changes in **A**: blood pressure, **B**: heart rate, **C**: heart rate variability, **D**: interventricular septum (IVS) in systole and **E**: left ventricular free wall (LVFW) in systole, with weight reduction for each dog achieving target weight reduction

Indirect SBP measurements for three dogs after weight reduction were missing. From the available data, there was no change in heart rate (*P* = 0.722, *r* = 0.117), HRV (*P* = 0.367, *r* = 0.271) or SBP (*P* = 0.674, *r* = 0.164), with weight reduction (Fig. 1). Cardiac biomarkers were missing for one dog after weight reduction; for the remaining 11 dogs, there was no significant change in hs-cTnI (*P* = 0.657, *r* = 0.147) or NT-proBNP (*P* = 0.262, *r* = 0.0.164) concentrations. For the one dog with an increased hs-cTnI concentration at baseline, this had normalised at second sampling post-weight reduction (0.033 ng/mL).

Of all the echocardiographic variables, only the systolic IVS and LVFW thickness changed with weight reduction (Table 2, Fig. 1): both reducing (IVSs median magnitude of change -1.4 mm, IQR -0.2 to -2.9, *P* = 0.029, *r* = 0.643; LVFWs median magnitude of change -1.8 mm, IQR -0.6 to -3.7, *P* = 0.017, *r* = 0.702). This was maintained when normalised to target bodyweight (IVSsN median magnitude of change -0.07, IQR -0.009 – 0.16, *P* = 0.011, *r* = 0.744; LVFWsN median magnitude of change -0.1, IQR -0.033 to -0.17, *P* = 0.009, *r* = 0.770). Despite an apparent significant change in end diastolic and end systolic volumes when indexed to actual bodyweight (EDVI, ESVI), this was not maintained when indexing to target weight, indicating this to be a direct consequence of the weight change, rather than an intrinsic change in volumes. There were no significant changes in any variables of diastolic or systolic function with weight reduction (Table 2).

### Daily sodium intake

When standardised to metabolic bodyweight, median sodium intake during weight reduction for all dogs was 65 mg per kg^0.75^ per day (IQR 64–76), and there was no difference between dogs that were fed different dry therapeutic weight loss diets despite their different sodium content (Satiety: 65 mg per kg^0.75^ per day, IQR 64–71; Satiety Small Dog: 71 mg per kg^0.75^ per day, IQR 64–79; *P* = 0.497, r = 0.196).

Median absolute daily sodium intake during weight reduction was 0.47 g per day (IQR 0.37–0.65 g per day). By comparison, the estimated daily sodium intake at maintenance were the same dogs fed a standard diet for neutered dogs would be 1.23 g per day (IQR 1.10–1.39 g per day). Therefore, sodium intake during weight reduction was estimated to be 51% (IQR: 26–59%) of the expected intake at maintenance (*P* < 0.002, r = 0.883).

## Discussion

Increased body fat is associated with increases in both cardiac preload and afterload which would be expected to affect both cardiac structure and function [13]. Such changes are well documented in people, but less so in dogs with obesity. Our study aimed to evaluate the effect of obesity and subsequent weight reduction in dogs on cardiac structure and function as assessed by echocardiography and cardiac biomarkers, as well as evaluating the effect on autonomic tone, assessed by heart rate variability. As far as the authors are aware, this is the first study to examine the effect of weight reduction on heart rate variability in dogs. Body composition results from DEXA confirmed significant weight reduction with reduction of fat mass rather than lean mass, as is the aim of a weight reduction regimen. Our study showed that dogs with obesity have signs of impaired diastolic function, which does not appear to improve with subsequent weight loss. However, a significant reduction in systolic left ventricular wall dimensions is seen in these dogs following controlled weight reduction, suggesting some of the cardiovascular changes seen with obesity may be reversible.

Development of LV hypertrophy in obesity is likely to be multifactorial; increased blood volume increases ventricular preload and wall tension, whilst systemic hypertension and increased peripheral resistance increase ventricular afterload, resulting in myocardial remodelling [13]. In the current study, LV wall dimensions were commonly above reference range in both diastole and systole before weight reduction; with increased diastolic IVS and LVFW, in 33% and 71% of dogs respectively at baseline, and increased systolic IVS and LVFW in 17% and 50% of dogs respectively. These results are similar to those reported in people with obesity, in which LV hypertrophy is commonly reported [4]. Our results are also concordant with those of Mehlman et al. [5], who reported increases in both systolic and diastolic wall thickness, but differ from those of Adolphe et al. [6] who reported that only LVFW-systole increased in obesity. The severity and duration of obesity prior to enrolment in the studies might have contributed to discrepancies between the studies. Wall thickness can also be affected by other factors including systemic hypertension, pseudohypertrophy due to volume depletion and heart rate.

Decreased LV hypertrophy with weight reduction is seen in people with obesity [8], and was also seen in this study, with a statistically significant reduction in systolic wall dimensions with weight reduction. When wall dimensions were normalised to the target body weight both pre- and post-weight-reduction (as opposed to actual bodyweight at each time point; allowing for a truer comparison between the two time points), the reduction in systolic measurements of both the interventricular septum and left ventricular free wall remained statistically significant. Neto et al. [10], reported a reduction in LVFW only, in both diastole and systole, and only in dogs that initially weighed > 30 kg. Piantedosi et al. [11] also reported a reduction in the diastolic IVS and LVFW following weight reduction. Adolphe et al. [6] also reported that LVFW decreased with weight reduction but, as with the current study, the systolic (but not diastolic) thickness improved. Therefore, it is likely that some degree of reverse remodelling of the LV with weight reduction can occur. Longer-term follow-up following weight reduction, may help to further explore this.

Diastolic dysfunction was seen in all dogs with obesity in the current study, systolic dysfunction being less frequently observed. Both diastolic and systolic dysfunction are reported to occur in people with obesity, resulting in an increased risk of heart failure [4, 13]. Development of diastolic dysfunction is multifactorial: triglyceride accumulation increases apoptosis of cardiomyocytes, RAAS activation and elevated aldosterone contribute to myocardial fibrosis, and elevated inflammatory cytokines contribute to fibrosis and increased wall stiffness, all contributing to diastolic dysfunction [4]. The current results are similar to a previous canine study [5]. That said, diastolic function can be affected by many other variables including age and BP, with diastolic dysfunction being a normal finding on echocardiography of older animals. Given the small sample size, we did not attempt to correct for these potentially confounding variables in the statistical comparisons. However, our cohort did include young and normotensive dogs in which diastolic dysfunction would not be expected, so we concluded that obesity was a more likely cause. An improvement in diastolic function with weight reduction, as reported in human literature [9, 20] was expected; however, no improvement was observed in our cohort. Possibly, a longer follow-up time post-weight reduction is required to see these changes, although this is speculative.

Obesity is associated with increased sympathetic drive [21] and, therefore, a decrease in HRV (which is an indicator of sympathetic tone) was expected; however, this was not seen. Whilst there are no published reference ranges for VVTI in dogs, our results were comparable to previous studies in dogs in ideal body condition [22], suggesting obesity did not affect the HRV in our cohort. We also hypothesised that weight reduction would result in increased vagal tone and, as a result, decreased heart rate and increased HRV; however, in contrast to our hypothesis, no changes in heart rate or HRV with weight reduction were seen. These results differ from studies in people [4] and from two previous canine studies where decreased HRV was seen in male dogs with obesity [16] and in mild to moderately overweight dogs [17]. A possible explanation for these differences is breed variation, since breed is known to affect HRV [22, 23]. Differences in methods to assess HRV likely also accounts for differences between studies. We used the time domain indicator, VVTI, which gives information about high frequency variation in heart rate, largely reflecting parasympathetic tone; however, circadian rhythm, blood pressure regulation, thermoregulation and RAAS activity may also affect the VVTI. Other studies, including that of Pongkan et al. [16] and Vieira et al. [17], have used a combination of both time and frequency domain analysis of HRV; the later study reporting a reduced HRV in overweight dogs only when using the high frequency index of HRV.

Cardiac biomarkers were within reference range for all but one dog in the current study, with no significant change following weight reduction. This could suggest that obesity was not contributing to clinically significant increased wall stress or myocardial injury. However, interestingly, in human heart failure patients with obesity a smaller increase in NT-proBNP occurs, compared to those with a normal body mass index [24], suggesting a more complex interaction between BNP and obesity.

Through increased sympathetic stimulation and RAAS activation, obesity increases blood volume, cardiac output and systemic vascular resistance, contributing to systemic hypertension in people [4, 25]. Therefore, hypertension was expected in our cohort, along with decreased BP following weight reduction and reduced sympathetic drive. Although increased SBP was observed in over half of the dogs in the current study, there was no significant change as a result of weight reduction. This corresponds to previously reported SBP findings in dogs with obesity; Aldolphe et al. [6] reported a clinically irrelevant increase in SBP in dogs with obesity, while Neto et al. [10], reported a significant reduction in SBP with weight reduction in dogs initially weighing > 30 kg, but not in other weight categories. Furthermore, Mooney et al. [26] reported no effect of BCS or body weight on SBP in 62 dogs, whilst Piantedosi et al. [11] reported no change in SBP in dogs with obesity following weight reduction. Although increased SBP was observed in a significant proportion of dogs in this study, this might not be related to obesity given the absence of significant SBP reduction following weight loss. Although the dogs were deemed to be clinically well, we cannot exclude the possibility of a non-identified comorbidity causing hypertension. More likely increased SBP might have been the result of stress whilst in the hospital (situational hypertension), rather than genuine hypertension, even though great care was taken to minimise stress and to acclimatise dogs prior to blood pressure measurement. Situational hypertension might account for why no decrease was seen as a result of weight reduction. Conversely, the lack of reduction in SBP with weight reduction in our study dogs, could be attributed to the focus on calorie restriction in the weight reduction regimen. In people with obesity and systemic hypertension, there is a multifaceted approach to treatment. Due to the role of sodium in regulation of extracellular volume, in addition to its direct effects on vasculature, dietary sodium restriction plays a key role in SBP reduction in people [27, 28]. The correct approach to dietary sodium in dogs with cardiac disease and/or systemic hypertension remains a debated topic [29]. Sodium was not purposefully restricted in our study; however, sodium intake when the study dogs were being fed therapeutic foods during weight reduction was within the National Research Council recommendations [30] and would likely be less than if fed a standard commercial diet for maintenance. Although we cannot fully exclude lack of salt restriction as a contributing factor for the persistent increase in SBP post weight-reduction, it seems unlikely that this was a contributing factor. Increased exercise also plays a role in managing systemic hypertension in people [31]. In our study, although lifestyle adjustments, including exercise modulation, were advised these were not strictly regulated, which may also have contributed to the lack of change in SBP.

The fact that we noted decreased systolic wall thicknesses with successful weight loss, but no significant change in blood pressure, indicates that the remodelling observed with the weight loss cannot merely be due to change in afterload or heart rate. This correlates to data in people with obesity showing that increased blood pressure and increased body mass index are independently associated with increases in left ventricular mass [32]. It is most likely that increases in wall thickness occur as a combined result of myocardial remodelling in response to altered afterload, in addition to myocardial and epicardial lipid deposition. The exact underlying mechanisms which result in the altered wall thickness and reduced diastolic function, across species, remain undetermined; mitochondrial dysfunction, increased production of reactive oxygen species, insulin resistance, leptin-resistance and intracytoplasmic lipid accumulation in cardiomyocytes all likely play a role in the cardiac changes seen with obesity [3, 32].

Obesity is a major risk factor for both morbidity and mortality in people. Obesity-related cardiac dysfunction is a major component of this, obesity being associated with an increased risk of cardiovascular death [4]. Considering the prevalence of obesity in the pet dog population worldwide, developing a better understanding of the effects of obesity on cardiovascular structure and function in dogs is of importance. Further study is needed to better understand the clinical consequences of the cardiac changes noted in canine obesity as reported in this study.

The main limitation of this work was the small number of study dogs. Firstly, this can lead to underpowering and inability to detect subtle changes in cardiac function. A power calculation to determine the required study population size was not performed, the number of dogs recruited was instead pragmatic, based on the number of cases likely to be recruited over the study time frame. However, our population size mirrored those in similar studies [5, 6, 10, 16, 17]. Due to the possibility of the study being underpowered to detect significant changes, the *P* value was not adjusted for multiple comparisons, which may itself be a further limitation of the study. Secondly, the small number of study dogs led us to use nonparametric tests, but by doing so the power to detect changes with weight reduction might have been further reduced. Thirdly, the small number of dogs in the study and limited echocardiographic differences before and after weight reduction meant we could not further explore possible associations between some of the DEXA and echocardiographic variables. Furthermore, there was a wide variation in the breed and size of dogs recruited, which might have reduced the ability to assess changes in HRV and added additional confounding variables. This being said, when assessing changes in cardiovascular variables with weight loss, each dog acted as its own control. Most cardiovascular variables can be affected by numerous factors which would act as confounding variables; diastolic function for example is highly affected by age, which was not controlled for in this study. However, as previously discussed, a wide age range was present in the study dogs, thereby making age-effects less likely (diastolic dysfunction would not be expected in younger dogs). Therefore, instead, obesity remains a likely causative factor for the findings. The use of age and breed matched, healthy controls, with normal BCS would have helped draw conclusions regarding the baseline data and changes seen due to obesity. Heart rate can affect some echocardiographic variables, such as the E deceleration time, which was not accounted for and may have acted as a confounding factor when making comparisons between pre and post weight reduction data, however such an impact should be minimal as there was no significant change in heart rate. A final limitation is that we did not perform follow-up echocardiography during the weight maintenance period, which might have helped assess for any further reverse remodelling or improved diastolic function.

## Conclusions

The results of the current study confirm that LV remodelling is a common echocardiographic feature in dogs with obesity, with evidence of reverse remodelling following weight reduction. We also demonstrated diastolic dysfunction to be a common finding in dogs with obesity, but no improvement with weight reduction was seen. To the authors’ knowledge, this is the first study to assess for changes in diastolic function and HRV with weight reduction. Contrary to our hypothesis, dogs with obesity did not have decreased HRV and, although systolic BP was frequently increased, no change with weight reduction was observed; this increase in SBP more likely reflecting situational hypertension than an effect of obesity. These results add to the current literature, that obesity in dogs has a cardiovascular impact and that some degree of reverse remodelling can be expected following a weight reduction regime. Follow-up of this cohort of dogs, following a period of sustained weight management may help to assess for more long-term effects of weight control.

## Methods

### Study animals

Dogs were referred to the Royal Canin Weight Management Clinic, University of Liverpool, for assessment and management of obesity. Cases were recruited between August 2016 and July 2017. To meet eligibility criteria, dogs could not have had significant cardiac or intercurrent disease, as assessed during initial examinations (see below). To be included in the final assessment, dogs had to have reached their weight reduction target by the study end date (April 2018). The study protocol was approved by the University of Liverpool Veterinary Research Ethics Committee (RETH000353 and VREC793) and the Royal Canin Ethical Review Committee (RCWMC_2021_01_V1). Owners of all participating animals gave written, informed consent.

### Weight reduction regimen

Details of the weight reduction programme have been previously described [33, 34]. In brief, dogs were determined to be clinically well with no systemic diseases that might affect the ability to achieve weight reduction (based on physical examination, haematology, serum biochemistry, urinalysis and free thyroxine performed during initial visit). Body condition score was assessed using a nine-point scale as previously described [1, 35]. At this initial visit, body composition was analysed by fan-beam DEXA, as previously described [36]. Briefly whole-body DEXA was performed under sedation, with dogs in dorsal recumbency, providing measurements of fat mass, lean mass and bone mineral content. These data were used to create individualised weight reduction plans for each dog, establishing both an ideal and a target weight (which considered age and severity of obesity). Briefly, this was achieved using a computer spreadsheet, containing a purpose‐created mathematical formula to predict expected body composition after weight reduction at different weights, based upon typical body composition results from previous weight clinic studies. These formulae were based on a predicted fat to non-fat mass loss of 80:20 [33], with the aim of reducing the body fat mass to within the reference interval for ideal body condition [33]. One of three commercially-available therapeutic diets were used for the controlled weight reduction protocol, dependent on breed size and also the preferences of both dogs and owners for wet and dry food (Supplement Table 3). Food allocation was estimated by calculating the metabolic energy requirement (MER = 440 kJ [105 kcal] x bodyweight [kg]^0.75^/day [30]) based on the ideal weight of the dog, as previously described [33]. Individualised advice on lifestyle and activity alterations were also given to assist in weight reduction by a registered veterinary nurse (GRTW).

Dogs were reweighed approximately every two to four weeks to assess progress, with subsequent changes to the weight reduction diet if required. This was performed either at the University of Liverpool (using the same, regularly calibrated, electronic scales) or, where logistics prevented this, at the dog’s primary care practice. Dogs were deemed to have reached the primary endpoint if target weight was achieved within the study period. Full laboratory analysis and DEXA were repeated at time of achieving target weight.

### Daily sodium intake

To assess dietary sodium intake, mean total daily sodium intake during weight reduction was calculated for each dog that completed weight management, and compared with the estimated daily sodium intake were the same dogs to be fed maintenance diets for neutered dogs (either Neutered Adult [sodium 0.3% as fed, metabolisable energy 3300 kcal per kg] or Neutered Adult Small Dog [sodium 0.8% as fed, metabolisable energy 3322 kcal per kg] as appropriate for size; both manufactured by Royal Canin). For these calculations, the metabolisable energy requirement for maintenance was assumed to be 95 kcal per kg^0.75^ [30]. Daily sodium intake during weight reduction, was also converted to intake per kg of metabolic bodyweight (kg^0.75^), to enable comparison with the minimum requirements and safe upper limit recommended of the National Research Council [30].

### Cardiac evaluation

Cardiac evaluation was performed prior to sedation for DEXA at both the initial visit and after target weight was reached. Dogs with pre-clinical MMVD were eligible, provided that it was not haemodynamically significant (i.e. only stage B1 MMVD [37]), considering the prevalence of such changes in an ageing population. Similarly, dogs with other asymptomatic, mild, primary cardiac disease including other trivial or mild valvular regurgitations were also eligible.

### Systolic blood pressure

Systolic blood pressure was measured indirectly by the Doppler method (Ultrasonographic Doppler Flow Detector 811-B; Parks Medical Electronics), as previously described [29]. Briefly, a cuff measuring 40% the circumference of the limb, was placed on a thoracic limb, with the dog sitting with the limb elevated to the level of the heart. Systolic blood pressure was measured in a quiet room with gentle handling prior to the other procedures. Dogs were allowed to acclimatise to the environment before five measurements were taken, with the mean value recorded. Values equal to and exceeding 160 mmHg were considered to be increased and consistent with systemic hypertension [29].

### Cardiac biomarkers

Blood was collected into EDTA tubes by jugular venepuncture at the initial and last assessments. Samples were immediately centrifuged and separated EDTA-plasma stored at -20 °C until after study completion and sent as a single batch on dry ice to an external laboratory (IDEXX Laboratories, Wetherby, West Yorkshire, UK) for measurement of hs-cTnI (Beckman Coulter Access hs-cTnI assay; IDEXX Laboratories) and second-generation NT-proBNP (Cardiopet proBNP test, IDEXX Laboratories).

### Electrocardiography

Six-lead ECG was obtained from all dogs, restrained in right lateral recumbency. Routine analysis of the ECG was performed, including rate, rhythm and standard lead II measurements. To calculate HRV, the R-R interval for 20 consecutive cardiac cycles was measured. The VVTI was then calculated as the natural logarithm of the variance of these R-R intervals (VVTI = Ln[SD_RR_]^2^) [38].

### Doppler echocardiography

Complete 2D, M-mode, colour flow and spectral Doppler echocardiography was performed with a Vivid 7 ultrasound machine (GE Healthcare), using a 4 or 7 MHz transducer. Procedures were either performed by an EBVS® European Veterinary Specialist in Small Animal Cardiology or a resident in training under the direct supervision of such a specialist. Echocardiography was performed without sedation, with dogs positioned in both right and left lateral recumbency. Simultaneous ECG was used for timing of events during the cardiac cycle. Analysis was performed on a remote, off-line measuring system.^1^ The mean value of three cardiac cycles, in sinus rhythm was obtained for each variable and used in analysis.

Standard echocardiographic views were acquired as previously described [39]. Endocardial-blood pool interface defined the boundaries for measurements on 2D echocardiography; the leading-edge-to-leading-edge method was used for M-mode measurements [40]. Left atrial diameter (LAmax) was obtained from the right parasternal long axis four chamber view; a right parasternal long axis five chamber view was used to measure the aortic (Ao) annulus systolic diameter, allowing calculation of the LAmax/Ao ratio [41]. The short axis left atrium to aorta ratio (LA/Ao) was measured from the right parasternal short axis view in early diastole [42]. M-mode of the LV was obtained from a right parasternal short axis view at the level of the chordae tendinae, with the cursor bisecting the LV cavity symmetrically. Non-normalised M-mode values were compared to breed reference ranges when available. For all dogs, allometric scaling was used to normalise LV dimensions to both the actual body weight and target bodyweight and published reference ranges used [18]. Modified Simpson’s rule was used to determine LV volumes in diastole and systole, from the right parasternal long-axis four-chamber view, optimising LV length and area. These end-diastolic and end-systolic volumes were normalised to both the actual body weight and target weight as mLs/kg (RI non-sight hounds: EDVI < 3.27 mL/kg; ESVI < 1.54 mL/kg) [43]. LV systolic function was assessed by Simpson’s derived ejection fraction (RI: > 50%). In addition, M-mode fractional shortening (RI: > 25%) was calculated using the standard formula [44]. Assessment of LV diastolic function included assessment of transmitral flow, measurement of E wave and A wave velocities, E wave deceleration time and A wave duration. Transmitral flow was obtained with the cursor sample volume between leaflet tips on a left apical four chamber view. From a left apical five chamber view, a sample volume was positioned between transmitral flow into the left ventricle, and left ventricular outflow, to enable measurement of the isovolumetric relaxation time (IVRT). Pulsed wave TDI was utilised to obtain myocardial velocities of longitudinal fibres at the septal and lateral mitral and tricuspid annuli (diastolic E’ and A’ and systolic S’ velocities) from a left apical four chamber view, ensuring alignment with each wall in turn. The following were considered to be markers of impaired LV relaxation: mitral E/A and TDI E’A’ < 1; prolonged IVRT (RI: > 54 ms); increased mitral E deceleration time (RI: 52–108 ms). If the transmitral E/A and IVRT were within reference ranges but TDI E’A’ < 1, this was considered pseudonormal diastolic function [44, 45]. Restrictive diastolic function is not defined here as no dog showed this (advanced cardiac disease excluded). Right ventricular systolic function was assessed by tricuspid annular plane systolic excursion (TAPSE) measured by M-mode with the cursor perpendicular to the tricuspid lateral annulus on a left apical view optimised for the right heart. For dogs that reached the end point, cardiac evaluations were repeated, allowing comparison between the two time-points.

### Statistics

Statistical analysis was performed with the use of commercially available software (Minitab, version 19). Sample size was based on pragmatic recruitment within the study time frame (rather than on power calculation). For every dog, a mean of each echocardiographic and clinical variable was recorded for each time-point. On account of the small sample size, the decision was made to use non-parametric tests. The median (and interquartile range) was reported for all descriptive statistics. Baseline weight, age and BCS were compared between the dogs that did and did not achieve weight reduction using a Mann–Whitney U test, and the same test was also used to compare daily sodium intake between dogs fed the different dry therapeutic diets. For the dogs that completed the study a Wilcoxon-signed rank test was used to compare each variable pre- and post- weight reduction, and the same test was used to compare daily sodium during weigh reduction and expected intake at maintenance on a standard diet. For LV wall thickness, comparison pre- and post-weight-reduction was made between the non-normalised values, as well as those derived from allometric scaling (both to the actual and the target bodyweights). Effect size was calculated as: *r* = Z / √N; *r* = 0.1 was considered small effect, *r* = 0.3 medium effect and *r* ≥ 0.5 large effect [46]. Missing data sets included DEXA for one dog, hs-cTnI for one dog at inclusion, cardiac biomarkers for one dog post weight reduction and SBP for three dogs post weight reduction, these were not accounted for statistically but were taken into consideration when interpreting the results. The level of statistical significance was set at *P* < 0.05, for two-sided analyses.

## Supplementary Information

**Additional file 1:** **Supplement Table 1.** Baseline demographic data for all enrolled dogs. Baseline data for all 24 enrolled dogs (sex, age, body weight, body condition score, blood pressure, heart rate, electrocardiographic and echocardiographic diagnosis)

**Additional file 2:** **Supplement Table 2.** Cardiovascular and echocardiographic variables for all 24 dogs at time of enrolment. Baseline echocardiographic and cardiovascular variables for all 24 dogs given as median and interquartile range.

**Additional file 3:** **Supplement Table 3.** Diet composition. Composition of the three commercially available weight management diets used in the weight management regimen.

## Data Availability

All data generated or analysed during this study are included in this published article [and its supplementary information files].

## Abbreviations

2D: Two dimensional
Ao: Aorta
AV: Atrioventricular
BCS: Body condition score
DEXA: Dual energy X-ray absorptiometry
ECG: Electrocardiography
EDVI: End-diastolic volume index
ESVI: End-systolic volume index
HRV: Heart rate variability
Hs-cTnI: High-sensitivity cardiac troponin I
IQR: Interquartile range
IVRT: Isovolumetric relaxation time
IVS: Interventricular septum
IVSsN: Systolic interventricular septum normalised to body weight
LA: Left atrium
LA/Ao: Short axis left atrium to aorta ratio
LAmax: Long axis left atrial diameter
LAmax/Ao: Long axis left atrium to aorta ratio
LV: Left ventricular
LVFW: Left ventricular free wall
LVFWsN: Systolic left ventricular free wall normalised to body weight
LVID: Left ventricular internal diameter
MMVD: Myxomatous mitral valve disease
NT-proBNP: N-terminal pro B-type natriuretic peptide
PW-TID: Pulsed wave tissue Doppler imaging
RAAS: Renin–angiotensin–aldosterone system
RI: Reference interval
SBP: Systolic blood pressure
TAPSE: Tricuspid annular plane systolic excursion
TDI: Tissue Doppler imaging
VVTI: Vasovagal tonus index

## Footnotes

1 GE Echopac version 113, GE Medical Systems, Buckinghamshire, UK.
