# Supplementary material for: The effect of obesity and subsequent weight reduction on cardiac structure and function in dogs
Source: BMC Vet Res. 2022 Sep 20;18:351. doi: 10.1186/s12917-022-03449-4 (PMC9487111; doi:10.1186/s12917-022-03449-4)
Supplement: Supplementary file 1 — Additional file 1: Supplement Table 1. Baseline demographic data for all enrolled dogs. Baseline data for all 24 enrolled dogs (sex, age, body weight, body condition score, blood pressure, heart rate, electrocardiographic and echocardiographic diagnosis) [file 12917_2022_3449_MOESM1_ESM.docx]

**Supplement Table 1:** **Baseline demographic data for all enrolled dogs.**

| **Dog** | **Breed** | **Sex** | **Age (years/months)** | **BW (kg)** | **BCS (/9)** | **Weight reduction achieved (Yes/No)** | **SBP** | **ECG HR** | **Echocardiography diagnosis** |
| --- | --- | --- | --- | --- | --- | --- | --- | --- | --- |
| 1 | Shih Tzu | FN | 10y2m | 11.4 | 6 | Y | 150 | 120 | Normal structure. Impaired relaxation |
| 2 | Small cross breed | FN | 6y5m | 12 | 7 | Y | 178 | 110 | Normal structure. Impaired relaxation |
| 3 | Small cross breed | FN | 4y7m | 10.1 | 8 | Y | 132 | 140 | Normal structure. Impaired relaxation |
| 4 | Rottweiler | FN | 7y8m | 39.4 | 7 | Y | 140 | 150 | Stage B1 MMVD and mild tricuspid valve degeneration |
| 5 | Bichon Frise | FN | 7y10m | 10.6 | 7 | Y | 220 | 110 | Stage B1 MMVD, first degree AV block, left anterior fascicular block |
| 6 | Cavalier King Charles Spaniel | FN | 7y6m | 14.4 | 8 | Y | 190 | 90 | Stage B1 MMVD. Impaired relaxation. |
| 7 | Cavalier King Charles Spaniel | FN | 11y2m | 14.2 | 8 | Y | 180 | 160 | Stage B1 MMVD |
| 8 | Bulldog | FN | 4y4m | 25 | 7 | Y | 180 | 120 | Hypertrophy of IVS |
| 9 | Border Collie | MN | 7y2m | 43.4 | 7 | Y | 184 | 110 | Normal structure. Impaired relaxation. |
| 10 | Border Collie | FN | 10y1m | 25.8 | 9 | Y | 160 | 60 | Normal structure. Decreased systolic function |
| 11 | Bulldog | FE | 1y8m | 26.1 | 9 | Y | 120 | 70 | Normal structure. Mild decreased systolic function |
| 12 | Newfoundland | ME | 3y5m | 67.0 | 8 | Y | 135 | 140 | Mild aortic stenosis (pressure gradient 26 mmHg). |
| 13 | Old English Sheepdog | ME | 8y9m | 41.8 | 7 | N | 140 | 140 | Stage B1 MMVD |
| 14 | Glen of Imaal Terrier | FE | 2y3m | 21.0 | 6 | N | 210 | 160 | Normal structure. Tachycardic. |
| 15 | Pug | FE | 2y10m | 11.1 | 9 | N | 144 | 100 | Normal structure |
| 16 | Cavalier King Charles Spaniel | MN | 4y10m | 12.3 | 8 | N | 142 | 120 | Mild decrease left ventricular internal dimensions. |
| 17 | Pug | FN | 3y6m | 7.4 | 8 | N | 150 | 90 | Normal structure |
| 18 | Pug | FN | 3y1m | 8.7 | 9 | N | 170 | 100 | Mild decrease left ventricular internal dimensions. |
| 19 | German Shepherd | MN | 4y6m | 76.0 | 9 | N | 200 | 80 | Mild decreased systolic function |
| 20 | Small cross breed | MN | 7y5m | 14.8 | 8 | N | 128 | 140 | Normal structure |
| 21 | Golden Retriever | MN | 8y7m | 56.0 | 9 | N | 172 | 140 | Stage B1 MMVD |
| 22 | Labrador | MN | 2y8m | 59.8 | 9 | N | 175 | 120 | Normal structure |
| 23 | Chihuahua | MN | 4y8m | 7.3 | 8 | N | 204 | 108 | Stage B1 MMVD |
| 24 | Jack Russell Terrier | MN | 9y5m | 11 | 8 | N | 160 | 140 | Mild left ventricular concentric hypertrophy. Stage B1 MMVD |

AV: atrioventricular, BCS: body condition score, BW: body weight, ECG: electrocardiography, FE: female entire, FN: female neutered, HR: heart rate, ME: male entire, MMVD: myxomatous mitral valve disease, MN: male neutered, IVS: interventricular septum, SBP: blood pressure,
