# Supplementary material for: The effect of obesity and subsequent weight reduction on cardiac structure and function in dogs
Source: BMC Vet Res. 2022 Sep 20;18:351. doi: 10.1186/s12917-022-03449-4 (PMC9487111; doi:10.1186/s12917-022-03449-4)
Supplement: Supplementary file 2 — Additional file 2: Supplement Table 2. Cardiovascular and echocardiographic variables for all 24 dogs at time of enrolment. Baseline echocardiographic and cardiovascular variables for all 24 dogs given as median and interquartile range. [file 12917_2022_3449_MOESM2_ESM.docx]

**Supplement Table 2:** **cardiovascular and echocardiographic variables for all 24 dogs at time of enrolment.**

| **Variable** | | | **Median** | **(IQR)** | **Reference interval** |
| --- | --- | --- | --- | --- | --- |
| **Body weight (kg)** | | | 14.60 | (11.03-41.20) |  |
| **BCS (/9)** | | | 8 | (7-9) | 4-5 |
| **ECG Heart rate** | | | 120 | (100-140) |  |
| **Heart rate variability (VVTI)** | | | 8.337 | (6.700-10.305) |  |
| **SBP (mmHg)** | | | 165 | (140-183) | <160 |
| **Hs-cTnI (ng/mL)** | | | 0.0090 | (0.0050-0.0150) | <0.070 |
| **BNP (pmol/L)** | | | 285.0 | (250.0-377.0) | <900 |
| **LA/Ao** | | | 1.28 | (1.13-1.38) | <1.5 |
| **LA major (cm)** | | | 3.18 | (2.51-3.97) |  |
| **LAmax/Ao** | | | 2.14 | (2.00-2.30) | <2.5 |
| **IVSd (mm)** | | | 9.65 | (7.95-12.25) |  |
| **LVIDd (mm)** | | | 30.45 | (26.13-43.17) |  |
| **LVFWd (mm)** | | | 8.90 | (7.73-11.08) |  |
| **IVSs (mm)** | | | 12.60 | (10.70-15.53) |  |
| **LVIDs (mm)** | | | 20.85 | (16.00-31.27) |  |
| **LVFWs (mm)** | | | 12.65 | (10.95-14.28) |  |
| **IVSdN** | **Actual weight** | | 0.40 | (0.37-0.46) | 0.27-0.49 |
|  | **Target weight** | | 0.45 | (0.40-0.51) |  |
| **LVIDdN** | **Actual weight** | | 1.25 | (1.17-1.35) | 1.17-1.63 |
|  | **Target weight** | | 1.41 | (1.30-1.50) |  |
| **LVFWdN** | **Actual weight** | | 0.42 | (0.36-0.46) | 0.30-0.53 |
|  | **Target weight** | | 0.47 | (0.41-0.51) |  |
| **IVSsN** | **Actual weight** | | 0.54 | (0.49-0.60) | 0.38-0.68 |
|  | **Target weight** | | 0.60 | (0.55-0.65) |  |
| **LVIDsN** | **Actual weight** | | 0.80 | (0.67-0.90 | 0.70-1.09 |
|  | **Target weight** | | 0.90 | (0.78-1.03) |  |
| **LVFWsN** | **Actual weight** | | 0.62 | (0.52-0.68) | 0.46-0.78 |
|  | **Target weight** | | 0.68 | (0.56-0.75) |  |
| **EDVI (mL/kg)** | | **Actual weight** | 1.49 | (1.25-1.70) | 1.25-3.27 |
| **ESVI (mL/kg)** | | **Actual weight** | 0.58 | (0.49-0.82) | 0.3-1.54 |
| **Mitral E velocity (m/s)** | | | 0.68 | (0.61-0.76) | <1.2 |
| **Mitral E/A** | | | 1.31 | (0.91-1.47) | 1-2 |
| **E deceleration (ms)** | | | 86.00 | (75.00-102.00) | 52-108 |
| **IVRT ms)** | | | 76.00 | (67.00-84.00) | 37-69 |
| **TAPSE (cm)** | | | 1.02 | (0.88-1.41) |  |
| **Mitral septal E/A** | | | 0.74 | (0.58-0.77) | 1-2 |
| **Mitral lateral E/A** | | | 0.71 | (0.57-0.80) | 1-2 |
| **Right lateral E/A** | | | 0.68 | (0.57-0.77) | 1-2 |
| **LVIDd/LVPWd** | | | 3.51 | (3.27-4.30) | 2.9-6.7 |
| **Fractional shortening (%)** | | | 32 | (28-39) | >25 |
| **Ejection Fraction (%)** | | | 60 | (52-66) | >50 |

EDVI: end diastolic volume index, ESVI: end systolic volume index, hs-cTnI: cardiac troponin I, suffix: -N: indexed to bodyweight, IQR: interquartile range, IVRT: isovolumetric relaxation time, IVSd: interventricular septum in diastole, IVSs: interventricular septum in systole, LA/Ao: left atrium: aorta ratio, LAmax/Ao: left atrium major to aorta ratio, LVFWd: left ventricular free wall in diastole, LVFWs: left ventricular free wall in systole LVIDd: left ventricular internal diameter in diastole, LVIDs: left ventricular internal diameter in systole, MV: mitral valve, NT-proBNP: N-type N-terminal pro-brain natriuretic peptide, SBP: blood pressure, TAPSE: tricuspid annular plane systolic excursion, VVTI: vasovagal tonal index
