# Supplementary material for: The effect of obesity and subsequent weight reduction on cardiac structure and function in dogs
Source: BMC Vet Res. 2022 Sep 20;18:351. doi: 10.1186/s12917-022-03449-4 (PMC9487111; doi:10.1186/s12917-022-03449-4)
Supplement: Supplementary file 3 — Additional file 3: Supplement Table 3. Diet composition. Composition of the three commercially available weight management diets used in the weight management regimen. [file 12917_2022_3449_MOESM3_ESM.docx]

**Supplement Table 3: Diet composition.**

|  | **Canine satiety Dry** | | **Canine Satiety Dry Small Dog** | | **Canine Satiety wet** | |
| --- | --- | --- | --- | --- | --- | --- |
| **Metabolisable energy content** | 2692kcal/kg | | 2669kcal/kg | | 247kcal/tin | |
|  | Per 100g | g/1000 kcal | Per 100g | g/1000 kcal | Per 100g | g/1000 kcal |
| **Protein** | 30.0 | 112.44 | 30.0 | 111.28 | 8.0 | 129.66 |
| **Crude fat** | 9.5 | 35.61 | 9.5 | 35.24 | 2.0 | 32.41 |
| **Crude fibre** | 17.0 | 63.72 | 15.6 | 57.86 | 2.0 | 32.41 |
| **Ash** | 5.8 | 21.74 | 6.8 | 25.22 | 1.0 | 16.21 |
| **Sodium** | 0.3 | 1.03 | 0.7 | 2.06 | 0.1 | 1.72 |

Composition of the three commercially available weight management diets used in the weight management regimen.
